# Assessing early erythrolysis and the relationship to perihematomal iron overload and white matter survival in human intracerebral hemorrhage

**DOI:** 10.1111/cns.13693

**Published:** 2021-06-17

**Authors:** Nemanja Novakovic, Zachary M. Wilseck, Thomas L. Chenevert, Guohua Xi, Richard F. Keep, Aditya S. Pandey, Neeraj Chaudhary

**Affiliations:** ^1^ Department of Neurosurgery University of Michigan Ann Arbor MI USA; ^2^ Department of Radiology University of Michigan Ann Arbor MI USA; ^3^ Department of Neurology University of Michigan Ann Arbor MI USA

**Keywords:** erythrolysis, hematoma, intracerebral hemorrhage, iron overload, magnetic resonance imaging, white matter loss

## Abstract

**Aims:**

Iron released from lysed red blood cells within the hematoma plays a role in intracerebral hemorrhage (ICH)‐related neurotoxicity. This study utilizes magnetic resonance imaging (MRI) to examine the time course, extent of erythrolysis, and its correlation with perihematomal iron accumulation and white matter loss.

**Methods:**

The feasibility of assessing proportional erythrolysis using T2* MRI was examined using pig blood phantoms with specified degrees of erythrolysis. Fifteen prospectively enrolled ICH patients had MRIs (3‐Tesla) at days 1–3, 14, and 30 (termed early, subacute, and late periods, respectively). Measurement was performed on T2*, 1/T2*, and fractional anisotropy (FA) maps.

**Results:**

Pig blood phantoms showed a linear relationship between 1/T2* signal and percent erythrolysis. MRI on patients showed an increase in erythrolysis within the hematoma between the early and subacute phases after ICH, almost completing by day 14. Although perihematomal iron overload (IO) correlated with the erythrolysis extent and hematoma volume at days 14 and 30, perihematomal white matter (WM) loss significantly correlated with both, only at day 14.

**Conclusion:**

MRI may reliably assess the portion of the hematoma that lyses over time after ICH. Perihematomal IO and WM loss correlate with both the erythrolysis extent and hematoma volume in the early and subacute periods following ICH.

## Graphical abstract

First study to quantify erythrolysis within hematoma in human intracerebral and correlate with perihematomal iron overload and white matter injury.

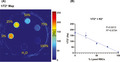

## 1 INTRODUCTION

The early and late prognoses in intracerebral hemorrhage (ICH) remain suboptimal.^1^, ^2^ Some nonmodifiable risk factors of ICH that dictate early prognosis include the anatomical location, size, and occurrence of intraventricular hemorrhage.^1^, ^3^ Hematoma expansion has been identified as a possible risk factor for poor outcome that can be potentially altered.^4^, ^5^ However, so far, targeting hematoma expansion has yet to definitively improve patient outcome.^6^ Similarly, attempts to significantly improve functional outcome with image‐guided hematoma evacuation following ICH have so far failed, although there is evidence of reduced mortality.^7^

More than two decades of basic science research in animal ICH models has identified iron as a major neurotoxin released from the hematoma after ICH.^8^, ^9^, ^10^, ^11^ However, those findings have yet to translate to patients. A lack of significant benefit in the deferoxamine phase II treatment trial in ICH^12^ suggests that our understanding of iron‐induced injury after ICH is still incomplete. Neuroimaging has a potentially significant role in unraveling some of the missing links in our understanding of the natural history of human ICH, particularly in relation to iron, given its paramagnetic properties. Magnetic resonance imaging (MRI) has been used to assess perihematomal iron following ICH.^13^, ^14^, ^15^, ^16^, ^17^ A pilot study using MRI demonstrated that hematoma volume impacts iron overload in the surrounding perihematomal tissue.^13^ Another parameter that may impact perihematomal iron after ICH is the rate of erythrolysis within the hematoma. The phenomenon of ultra‐early erythrolysis has been demonstrated in animal ICH models and in human ICH on MRI.^11^, ^18^, ^19^

The aims of the current study were to determine whether MRI can be used to quantify erythrolysis in the hematoma in human ICH, evaluate the time course of erythrolysis, and examine whether the extent of erythrolysis correlates with perihematomal iron overload and white matter loss. No such analysis has been performed to date, and our study is part of a larger focus on developing novel imaging prognostic markers for ICH.

## 2 METHODS

### 2.1 Institutional approval and patient selection

The University of Michigan Institutional Review Board approved the study, and all patients consented to be included. Prospective patient recruitment commenced in early 2013 to acquire pilot data to translate the understanding of the ICH natural history from animal ICH models to human subjects. The pilot project with 3 patients and one control (normal human subject) led to successful application to the National Institutes of Health with secured funding in terms of two R21 grants in 2017 and 2018. Until the beginning of 2020, a total of 15 patients were recruited. All patients with basal ganglia hemorrhage were included, and MRI was performed on days <3, 14, and 30 following the hemorrhage. Exclusion criteria were previous hemorrhage or calcification in the basal ganglia region on noncontrast head CT and any contraindication for performance of MRI scans. Fifteen patients recruited to the study were analyzed. Not all patients had MRIs performed on all the prespecified data points, and not all screened and eligible patients were included in the study due to refusal to join.

### 2.2 Magnetic resonance imaging

Brain MRI examinations were performed on a 3T MRI system using a 32‐channel head coil for acquisition of standard 3D T1‐weighted, T2‐weighted, and fluid‐attenuated inversion recovery sequences. White matter integrity was probed using 32 direction b‐values 0–800 s/mm^2^, single‐shot echo planar diffusion tensor imaging for generation of fractional anisotropy (FA) maps along with other diffusion/anisotropy metrics at 1 × 1 × 2.3 mm^3^ resolution. Relaxivity maps, sensitive to the presence and distribution of iron, were generated from a 3‐dimensional (3D) eight‐echo gradient echo scan at following timings: TR = 40 ms and TE = 6.5 ms + (n‐1) × 4.5 ms, where n = 0, 1,…, 7. Quantitative maps R2* (in Hertz units) and T2* (in millisecond units) at a pixel resolution 0.83 × 0.83 × 1.5 mm^3^ were generated by monoexponential fit to pixel signal decay versus TE.

### 2.3 Examination of erythrolysis within the hematoma

**FIGURE 1:**
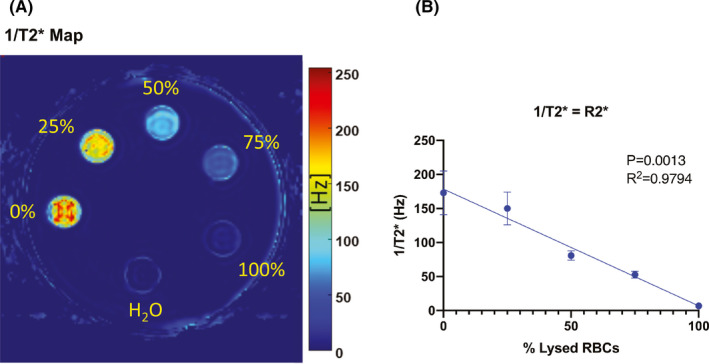
Porcine blood phantom vials containing different percentages of lysed red blood cells (RBCs) and one vial with purified water were evaluated using T2* MRI. T2* maps were created, and 1/T2* = R2* (Hz) values determined. A, An example of such analysis, with color‐coded image for 1/T2*. The percentage of cell lysis is shown against each vial. B, A plot of 1/T2* against the percentage of lysed RBCs shows a linear relationship (*p* = 0.0013, R^2^ = 0.979). Values are represented as means ± standard deviation, *n* = 6

For each subject and time point, a volume of interest (VOI) sphere was manually defined on all slices along the hematoma boundary on 1/T2* maps. A custom analysis routine written in MATLAB (version R2015b; MathWorks^®^) was used to calculate the “non‐hypointensity” volume within the hematoma as follows. A display of the slice containing the greatest hematoma cross‐sectional area based on the VOI mask was used to prompt manual definition of a circular contralateral region (nominal area ~425 mm^2^) in the normal brain on the first‐echo (TE = 6.5 ms) image. A circle of interest at each slice was automatically collated into a sphere of interest including all the slices of interest showing the hematoma. An identical sphere of interest was applied to the contralateral anatomically identical location. Calculation of (mean – standard deviation) for pixels within the contralateral region on the first‐echo image was used as a “reference” signal. The measurement on the contralateral basal ganglia region, considered to be normal, was deemed a value of 100% signal. The threshold was chosen as 1 standard deviation below the basal measurement on the contralateral side. Anything at or above this threshold was deemed to be a non‐hypointense signal within the measured hematoma volume. Pixels on the first‐echo 3D volume within the hematoma VOI having a value greater than the reference signal were summed to estimate “non‐hypointensity” volume for the hematoma. A porcine blood phantom was utilized to test the hypothesis of lack of susceptibility from lysed red blood cells (RBCs). Fresh porcine blood from a euthanized swine was collected. Packed RBCs were obtained by centrifuging unclotted blood. The plasma and buffy coat were discarded. The RBCs were washed with five volumes of saline three times. To prepare lysed RBCs, packed RBCs were frozen in liquid nitrogen for 5 min, followed by thawing at 37℃. Various proportions of lysed and unlysed blood were prepared in 4‐mL vials. These were then stuck on the undersurface of the lid of the water bath phantom. Pure nonionic water was utilized to prepare the water bath. This phantom was then scanned in a 3T MRI scanner with a protocol identical to the one applied for humans. Maps of 1/T2* were then created and analyzed (Figure 1). The authors hypothesize that the MRI neutral environment of the water bath mimics the brain parenchyma in human ICH. Moreover, the contralateral normal anatomy was used to normalize the data measured within the hematoma and its surrounding tissue on the side of the ICH.

### 2.4 Perihematomal iron overload

Perihematomal iron overload was determined, as described previously.^17^ In brief, on MRI phantoms, there is a linear relationship between R2* and iron concentration.^13^ Therefore, MATLAB files for R2* images for each patient were created, and three concentric rings (2 mm thick) were drawn around the hematoma, which were used to calculate iron concentration. Control iron was calculated using contralateral basal ganglia. The total iron overload (milligrams) was calculated by comparing ipsi‐ and contralateral iron concentrations multiplied by the volume of the rings: iron overload = ((ring 1 [Fe]‐contr [Fe]*(ring 1 volume)) + ((ring 2 [Fe]‐contr [Fe]*(ring 2 volume)) + ((ring 3 [Fe]‐contr [Fe]*(ring 3 volume)).

### 2.5 Perihematomal white matter loss

White matter loss was assessed using FA maps of two spheres, one centered on, but excluding, the hematoma (henceforth called perihematomal or perilesional tissue) and a second mirror contralateral sphere. The radii of the spheres ranged between 2.0 and 2.5 cm. FA is heavily dependent on the unidirectionality of white matter fibers, and voxels with an FA volume >0.5 were considered to be relatively healthy white matter. This arbitrary figure was used to lessen bias in determining white matter volume. The individual voxels were used to determine the amount of white matter within the perihematomal tissue and the contralateral sphere. The contralateral value was used to estimate an expected volume of white matter in the perihematomal tissue. ICH‐induced white matter loss (in milliliters) was determined as the difference between that number and the measured perihematomal white matter volume.

### 2.6 Statistics

The relationships between erythrolysis and other imaging parameters were examined by regression analysis. Comparisons between means were conducted by using analysis of variance (ANOVA) if the data passed a test of normality (Kolmogorov‐Smirnov test). For non‐normal data, Mann‐Whitney and Wilcoxon nonparametric tests were employed (as indicated in text). Statistical significance was taken as *p* < 0.05. This was a pilot study, and a power analysis was not performed.

## 3 RESULTS

MRIs on pig blood phantoms demonstrated a graded decrease in susceptibility (i.e., bright signal) as the blood sample in the vial approached 100% lysis of RBCs (*p* = 0.0013, R^2^ = 0.979). The graded decreased T2* signal (non‐hypointensity) is shown in the color‐coded MRI and the graph in Figure 1. This suggests the feasibility of using MRI to examine the degree of erythrolysis within a hematoma after ICH.

A total of 15 patients aged 22 to 84 years were recruited for the study. They had data points collected at post‐bleed days <3, 14, and 30, although not all data points were collected in each patient (Table 1). Thus, three patients had an MRI performed at day 1, thirteen patients had an MRI performed at day 3, and ten patients had data points collected at both days 14 and 30. Incomplete datasets were the result of loss to follow‐up and patient family refusal of continued study participation. The patient's neurological status at presentation was recorded and so was the functional outcome at the latest follow‐up, with good documentation of functional assessment in the patient's medical records. The majority of small hematomas made a good recovery to an mRS (modified Rankin score) of 0–2. Two patients with large‐sized hematomas above 25 mL had mRS scores of 3 or 4 at the last follow‐up (range 4 months to 2 years 54 days, Table 1). There is wide variation in clinical recovery of patients in terms of motor function. Although scientific determination cannot be performed with such a small number of patients, the authors hypothesize that the extent of erythrolysis and variations in individual iron handling capabilities in patients could have bearing on the degree of white matter recovery in the perihematomal tissue.

**TABLE 1:** Magnetic resonance imaging data and patient results.

Patient	Age (year)/gender	Magnetic resonance imaging (MRI) analyses					Hematoma	Motor examination at onset	Motor examination at the last follow‐up (time since onset)	mRS at the last follow‐up
		MRI day post‐bleed	Iron overload	Edema	Erythrolysis volume	Perilesional FA	Size, T2* (mL) (day)			
01	69 / F	14 30	X X	X	X X	X X	39.0 (14)	LUE and LLE 0/5	LUE and LLE 2/5 (11 months)	4
02	51 / M	14 30	X X	X	X X	X X	2.4 (14)	LUE and LLE 4+/5	LUE and LLE 5/5 (2 years, 64 days)	1
03	48 / F	3 14 30	X X X	X X	X X X	X X X	8.9 (3)	RUE and RLE 0/5	RUE and RLE 5/5 (16 months)	1
04	55 / M	3 14 30	X X X	X X	X X	X X X	0.31 (3)	UE and LE 5/5	UE and LE 5/5 (4 months)	1
05	69 / M	3 14 30	X X X	X X	X X X	X	5.2 (3)	LUE 0/5, LLE 1/5	LUE 1/5, LLE 5/5 (6 months)	3
06	25 / M	3 14			X X		36.6 (3)	RUE and LE 5/5	RUE and RLE 5/5 (8 months)	0
07	63 / F	1 3	X X	X X	X X	X X	11.5 (1)	RLE 4/5	RLE 5/5 (8 months)	0
08	23 / F	3 14 30	X X X	X X	X X X	X X X	18.9 (3)	RUE and RLE 0/5	RUE 4/5, RLE 5/5 (56 days)	2
09	39 / F	3 14 30	X X X	X X	X X X	X X X	5.5 (3)	LUE 4/5, LLE 5/5	LUE and LLE 5/5 (60 days)	0
10	72 / F	3	X	X	X	X	2.1 (3)	UE and LE 5/5	UE and LE 5/5 (75 days)	0
11	84 / F	3 14 30	X X X	X X	X X X	X X X	6.6 (3)	LUE 4/5, LLE 5/5	LUE 5/5, LLE 4/5 (120 days)	2
12	64 / M	3 14	X	X	X	X X	15.0 (3)	UE and LE 5/5	UE and LE 5/5 (4 months)	1
13	65 / F	1 3 14 30	X X X X	X X X X	X X X X	X X X X	10.9 (1), 10.8 (3)	LUE 3/5, LLE 4/5	LUE and LLE 5/5 (2 months)	1
14	57 / F	3	X	X	X		17.5 (3)	LUE and LLE 0/5	LUE and LLE 5/5 (7 months)	0
15	66 / M	1 3 14 30	X X X X	X X X X	X X X X	X X X X	13.8 (1), 12.6 (3)	LUE and LLE 0/5	LUE 0/5, LLE 2/5 (8 months)	4

Note

All hematomas were basal ganglia, except for Patient 06, who had a left‐sided parasagittal hematoma +IVH.

Abbreviations: FA, fractional anisotropy; LE, lower extremity; LLE, left lower extremity; LUE, left upper extremity; mRS, modified Rankin Scale score; RLE, right lower extremity; RUE, right upper extremity; UE, upper extremity.

**FIGURE 2:**
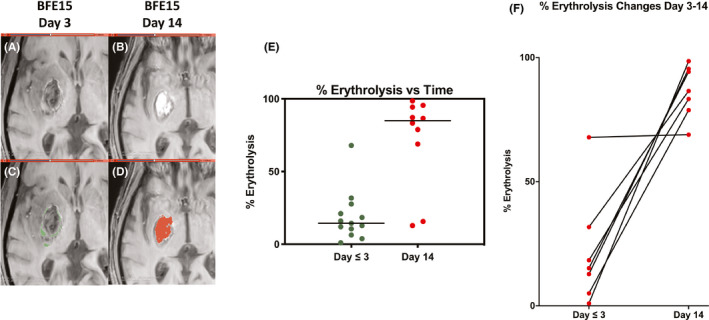
Sixty‐seven‐year‐old male patient presenting with 25‐mL right putaminal ICH. Axial T2* imaging was performed at days 3 (A) and 14 (B). These show the difference in the extent of T2* non‐hypointense signal within the hematoma with time. Non‐hypointense regions of ICH (depicting erythrolysis volume), shown by the shaded regions at days 3 (C; green) and 14 (D; red), demonstrate increased erythrolysis volume with time post‐ICH presentation. E, The non‐hypointense T2* volume was divided by hematoma volume to calculate percentage erythrolysis in individual patients examined at days 1–3 and 14. The percentage erythrolysis increased markedly between those time points (Mann‐Whitney *p* = 0.0006; *n* = 10–13). F, Almost all patients examined at both days ≤3 and 14 showed a marked increase in percent erythrolysis with time (Wilcoxon *p* = 0.0156; *n* = 7)

**FIGURE 3:**
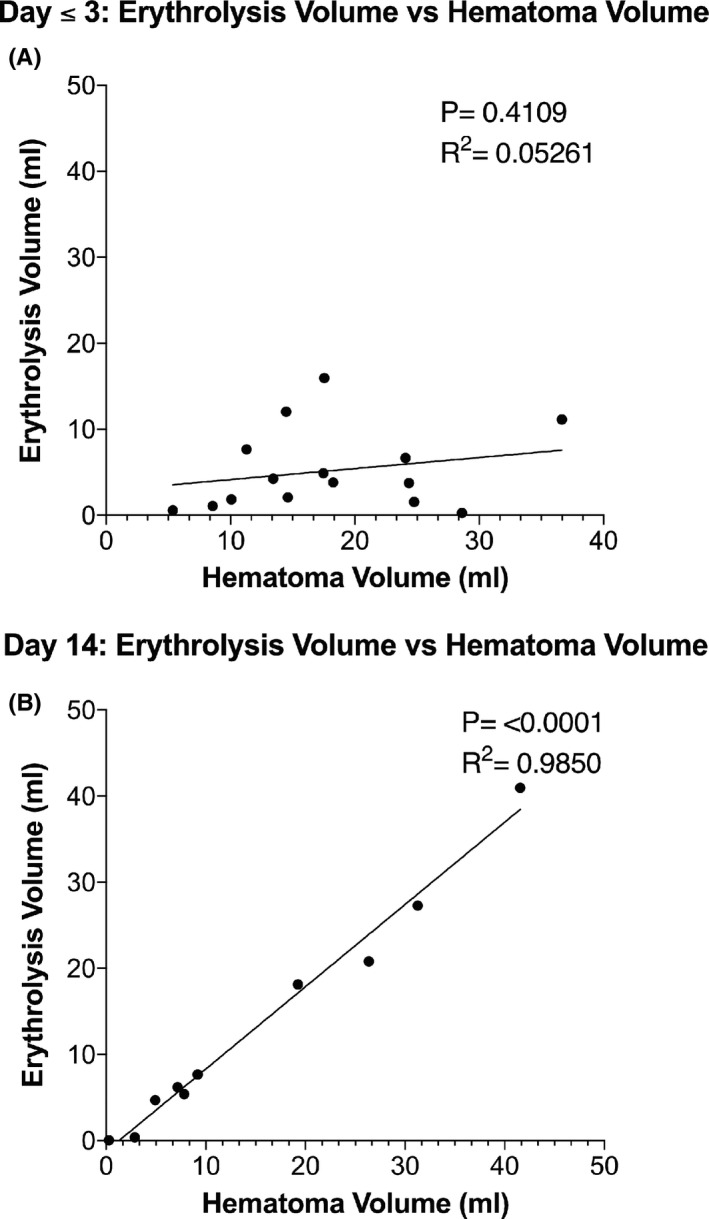
Relationship between erythrolysis and hematoma volume at days (A) ≤3 and (B) 14. There was a highly significant correlation between erythrolysis volume and hematoma volume at day 14 (*p* < 0.0001, *R* ^2^ = 0.985), but not for day ≤3 (*p* = 0.4109, R^2^ = 0.053)

Examples of the MRI T2* sequence used to determine percent erythrolysis within the hematoma are shown in Figure 2A (day 3) and Figure 2B (day 14); note how the appearance of the hematoma on MRI changes with time. The determined erythrolysis areas on those scans are shown in Figure 2C (day 3, green) and Figure 2D (day 14, red) and quantified in Figure 2E. As seen in the latter, there is a marked increase in the percent erythrolysis between patients examined at day 1 or 3 after ictus and day 14 (Mann‐Whitney *p* = 0.0006), although there are outliers, with one patient showing marked early erythrolysis and two showing less delayed erythrolysis. Almost all patients examined at both day ≤3 and day 14 showed a marked increase in percent erythrolysis with time (Figure 2F, Wilcoxon *p* = 0.0156; *n* = 7). Figure 3 shows how percent erythrolysis changed between day 1 or 3 and day 14 in patients with scans at each time frame. As can be seen, almost all patients showed a marked increase with time apart from the patient with marked early erythrolysis. The relationship between absolute hematoma and erythrolysis volumes was examined. At day 1 or 3, there was no significant correlation (Figure 3A). In contrast, at day 14, there was a very tight correlation (*p* < 0.0001, *R* ^2^ = 0.9850; Figure 3B).

**FIGURE 4:**
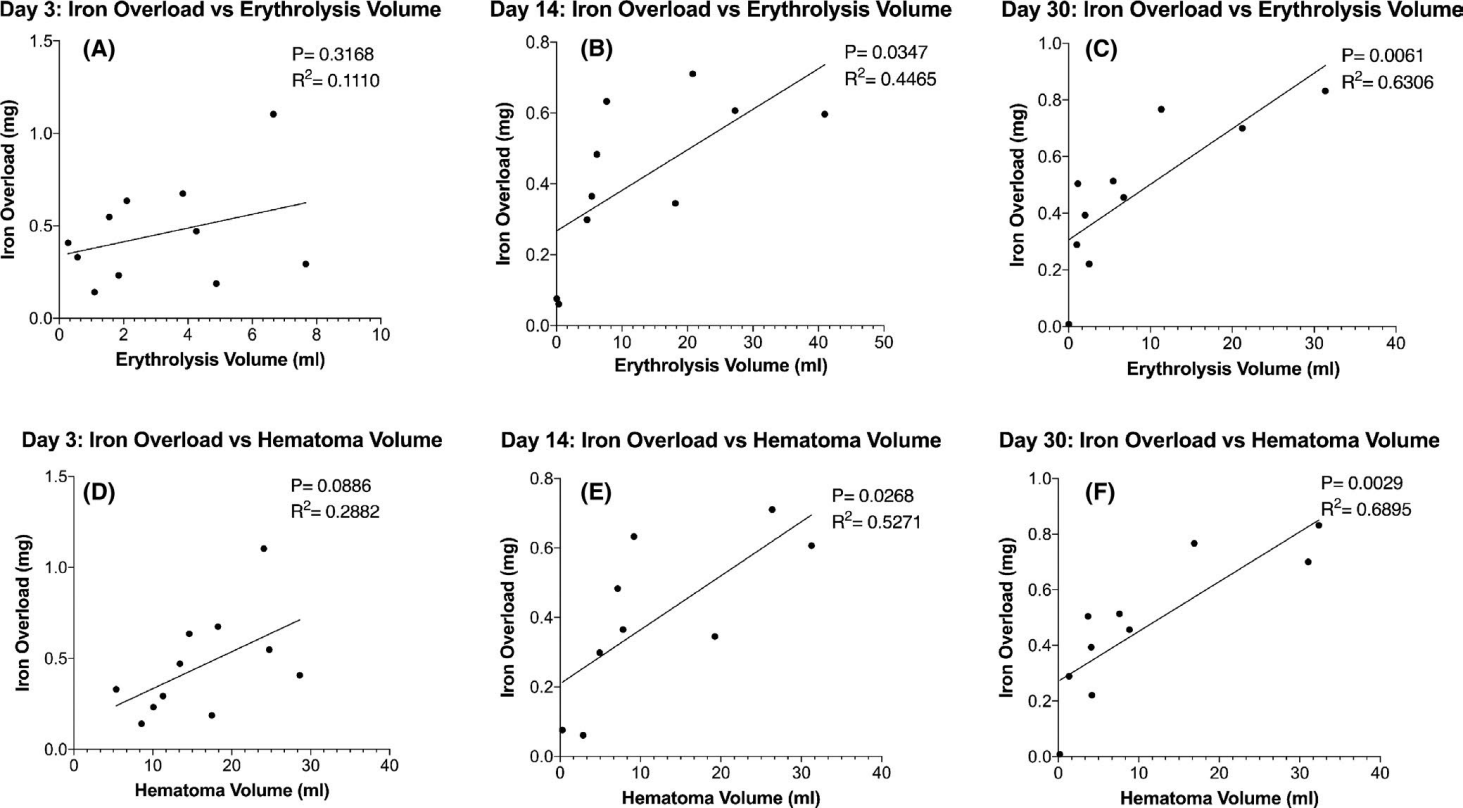
Relationship between perihematomal iron overload and erythrolysis volume at days 3, 14, and 30 is shown in the top graphs (A‐C). There was a significant relationship at days 14 (*p* = 0.0347, *R* ^2^ = 0.447) and 30 (*p* = 0.0061, *R* ^2^ = 0.631), but not at day 3 (*p* = 0.3168, *R* ^2^ = 0.1110). Bottom graphs (D‐F) show the relationship between iron overload and hematoma volume. There was a significant relationship at days 14 (*p* = 0.0268, *R* ^2^ = 0.527) and 30 (*p* = 0.0029, *R* ^2^ = 0.690), but not at day 3 (*p* = 0.0886, *R* ^2^ = 0.288)

Perihematomal iron overload was calculated based on the difference in iron concentration between the ipsilateral and contralateral tissues and the volume of tissue involved (see Methods). Iron overload and erythrolysis volume, as assessed on R2* and T2* images, respectively, were calculated at days 3, 14, and 30. Perihematomal iron overload ranged from 0.008 to 1.4 mg. At days 14 and 30, there was a positive correlation between iron overload and erythrolysis volume. Hematoma volumes were also assessed at days 3, 14, and 30. Hematoma volumes ranged from 0.2 to 42 mL. At days 14 and 30, there was a positive correlation between iron overload (milligrams) and hematoma volume (milliliters) (*p* = 0.0268, *R* ^2^ = 0.527 and *p* = 0.0029, *R* ^2^ = 0.690) (Figure 4).

**FIGURE 5:**
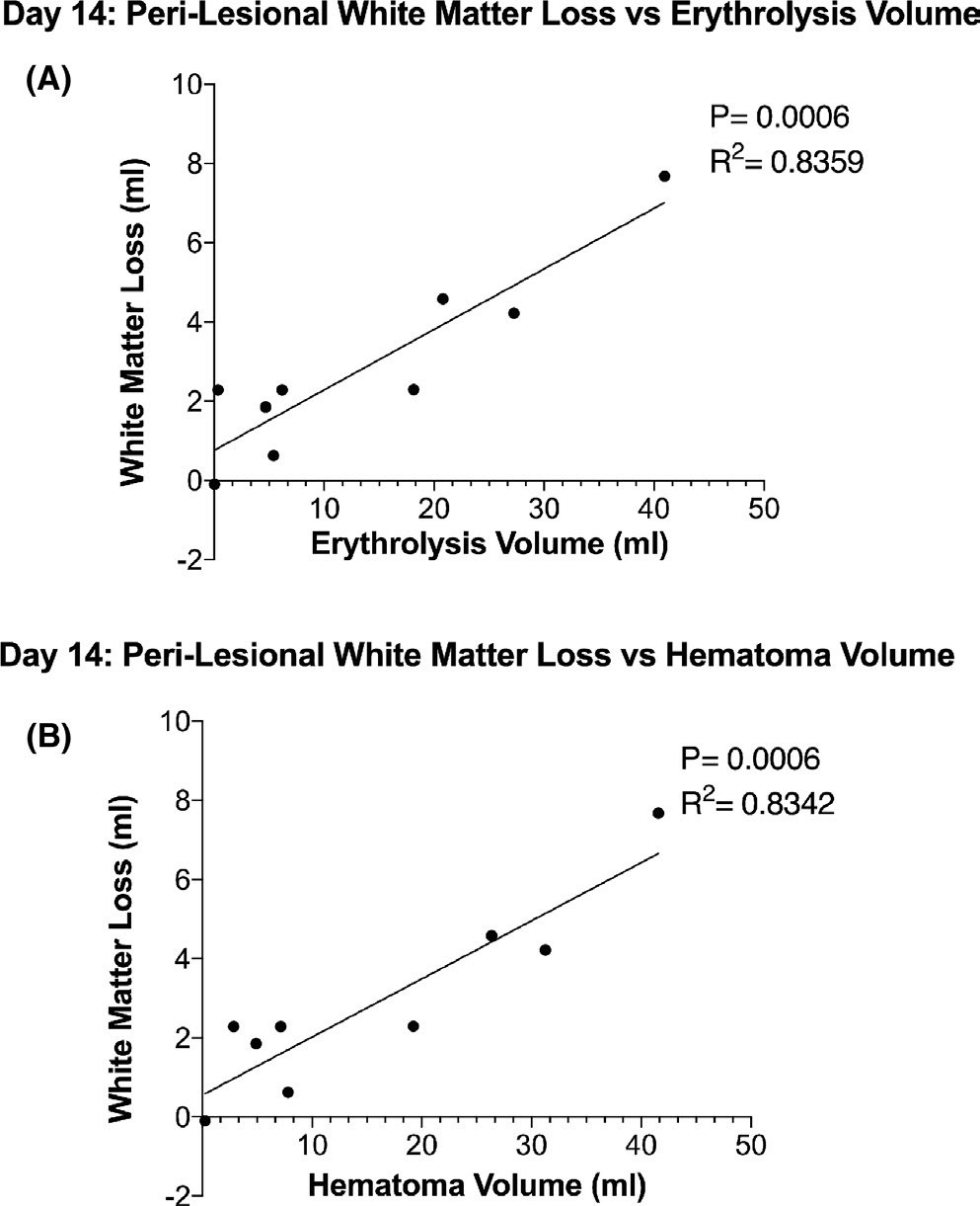
Relationship between perilesional white matter loss and either erythrolysis volume (A) or hematoma volume (B) at day 14. There was a significant correlation with perilesional white matter loss at day 14 with both erythrolysis volume (*p* = 0.0006, *R* ^2^ = 0.836) and hematoma volume (*p* = 0.0006, *R* ^2^ = 0.834)

Perihematomal white matter loss was calculated and based on fractional anisotropy values >0.5 using the contralateral side as an internal control (see Methods). At day 14, the amount of perilesional white matter loss ranged from 0 to 4.6 mL. There was a positive correlation between perilesional white matter loss and both erythrolysis volume (*p* = 0.0006, *R* ^2^ = 0.836; Figure 5A) and hematoma volume (*p* = 0.0006, *R* ^2^ = 0.834; Figure 5B).

## 4 DISCUSSION

This study has several major findings. (1) Pig blood phantoms show that T2* imaging can be used to quantify the degree of erythrolysis. (2) In ICH patients with basal ganglia hemorrhage, the percentage erythrolysis within the hematoma progressively increases from ≤3 days to day 14. (3) The erythrolysis volume and hematoma volume correlate well at day 14 but not at day ≤3. (4) There is good correlation of erythrolysis volume and perihematomal iron overload at days 14 and 30, but not at day 3. Similarly, there is good correlation between the hematoma size and iron overload at days 14 and 30, but not at day 3. (5) Very interestingly, there is an excellent relationship between the erythrolysis volume and hematoma volume with white matter loss at day 14.

There is wide variation in clinical recovery of ICH patients in terms of motor function. Although scientific determination cannot be performed with such a small number of patients, the authors hypothesize that the extent of erythrolysis up to 14 days following ICH and variations in individual iron handling capabilities in patients could have bearing on the degree of white matter recovery in the perihematomal tissue. Measurements of these parameters on MRI are novel but readily acquired (as demonstrated in this study) and could inform to prognosticate individual patient's motor function recovery. This aspect has not been studied to date, and the authors are encouraged by these findings to embark on studying its validity in a large population of human ICH patients.

Early erythrolysis has been noted in a preclinical rat ICH model with the appearance of areas within the hematoma with “ghost” erythrocytes as demonstrated by histology.^18^ This was correlated with the appearance of non‐hypointense areas within the hematoma on T2* MRI.^18^ Similar non‐hypointense areas within hematomas were noted in human ICH patients on T2* MRI^19^ and postulated to reflect ultra‐early erythrolysis. To investigate this further, the current study used MRI phantoms of pig blood with defined degrees of erythrolysis. Those phantoms showed an excellent correlation between percent erythrolysis and R2* (1/T2*) signal (Figure 1). We, therefore, suggest that regions of T2* non‐hypointensity within the hematoma can be a surrogate marker for early erythrolysis. We propose that hemoglobin‐bound iron loses its ability to create susceptibility in the electromagnetic field of the MRI after cell lysis (and hemoglobin dispersion). Thus, the MRI T2* maps show a bright to isointense signal and not a typical dark signal from iron and its ensuing susceptibility. Whether any phenomenon other than erythrolysis can cause such a change in the T2* signal within hematomas needs to be investigated. However, our study is the first of its kind to quantifiably translate the benchside understanding of ultra‐early erythrolysis in small animal ICH models (“ghost RBC” shown on histology), demonstrating it in a porcine (large animal) blood phantom and then in ICH patients.

In the current study, the degree of erythrolysis in the hematoma increased markedly between the early (days 1 and 3) and subacute (day 14) phases, being almost complete in the latter. However, there were outliers where erythrolysis was faster or slower. It is worth noting that patients with similar hematoma sizes and locations can have very different outcomes. Clinical analysis of the patients recruited in the study demonstrated that although large hematoma sizes of about 40 mL had poor outcomes, with an mRS of 4 at their latest follow‐up, the midrange of hematoma sizes (10–20 mL) had varied outcomes from intubation at the onset of ICH to full recovery at 90 days to 5–6 months or an mRS of 2–3 (Table 1). Whether different rates of erythrolysis might contribute to such variation merits further investigation. However, it will require a much larger sample than that in the current study.

Erythrolysis volume was positively correlated with the amount of perihematomal iron accumulation at days 14 and 30 but not at the early phase (day ≤3). The correlation at the two later time points presumably reflects the importance of erythrocytes as the source of perihematomal iron. We postulate that the lack of a correlation at the early time point may reflect a balance between how quickly iron (hemoglobin) released after erythrocyte lysis permeates into the surrounding tissue or the effects of mopping up mechanisms in clearing initial iron release from the hematoma. It should be noted that hematoma volume also only correlates with perihematomal iron overload at days 14 and 30, and not during days ≤3. The current study did not have enough patients of similar hematoma sizes to examine whether variations in the rate of erythrolysis impact the amount of perihematomal iron overload. No other analysis has looked into the association of the degree of erythrolysis and iron overload. Our study is the first to demonstrate this association.

The current study showed areas of hemolysis within the cores and peripheries of hematomas. The factors determining the rate of erythrocyte lysis within a hematoma have still to be fully determined. It may depend on erythrocyte energy depletion (which might favor lysis in the core) as well as the migration of factors from the perihematomal tissue and its blood supply. Infiltrating macrophages and resident microglia play a role in hematoma resolution.^20^ In addition, the complement system, via membrane attack complex activation, also plays a role in erythrocyte lysis.^11^

Erythrolysis volume and hematoma volume both correlated with white matter loss in the perihematomal tissue on day 14. This sort of correlation has not been investigated before in the perihematomal region, and the authors hypothesize that it is a novel method of examining the impact of ICH on perihematomal white matter fibers in the basal ganglia region. Erythrolysis volume and hematoma volume showed similar correlations to white matter loss (*R* ^2^ = 0.836 and 0.834, respectively). Again, there were very few patients to examine whether variations in the speed of erythrolysis impacted white matter loss. Recently, complement inhibition was shown to attenuate both early erythrolysis and brain injury in a rat ICH model.^11^ It would be important to examine if such an approach would also reduce perihematomal white matter loss in a gyrocephalic species.

The current study used novel methods to assess the change in erythrolysis in the hematoma and its relationship with perihematomal iron overload and white matter loss. In order to avoid bias in particular, the contralateral tissue was used to provide reference values in each of the measurements. Thus, the contralateral tissue was used as baseline to define what was non‐hypointense for the erythrolysis measurement, contralateral iron measurements were used to calculate iron overload, and contralateral white matter volumes were used to estimate white matter loss. The latter is very important considering anatomical variations in white matter density. As explained in the Methods section, FA maps were created from diffusion tensor imaging sequences acquired on MRI. This technique of white matter evaluation has not been performed before. Our study demonstrates the use of MRI sequences used routinely in everyday clinical practice and hence with a potential for a more universal applicability once established in the future. The cross‐referencing with a threshold of 0.5 FA value is novel as this strategically includes white matter fibers on the affected side, which may not be completely healthy and have FA values of 0.8 or 0.9 as in areas of normal densely packed white matter tracts.^21^

Currently, CT‐based assessment of hematoma size and surrounding edema lacks granularity.^22^ We suggest that MRI‐based assessment provides a more robust assessment of various markers of tissue toxicity (perihematomal iron overload and white matter loss) in the perihematomal region but also can be correlated to cellular events such as erythrolysis occurring within the hematoma and the hematoma size itself. It is encouraging to find the temporal trends toward peak effects of erythrolysis by day 14 of ICH can be reliably tracked.

Our study has several limitations, most important being the small number of patients. The analysis is also limited by the lack of MRI on each recruited patient at all the time points of the study (days 1, 3, 14, and 30). A larger number of patients in the cohort with similar sized hematomas are needed to analyze variations in the extent of erythrolysis that occurs within different hematomas. Such a larger study could also be used to examine whether the degree of erythrolysis correlates with functional outcome.

## 5 CONCLUSION

Our study shows that multiparametric assessment of the hematoma and the surrounding tissue by MRI could provide tissue injury markers for a more informed assessment of extent of ICH‐induced damage. Assessment of hematoma volume and erythrolysis volume within the hematoma may indicate the severity of the toxic onslaught inflicted on the surrounding brain tissue. Tracking these parameters on MRI over a month might provide insights into peaks of erythrolysis within the hematoma and iron overload on the surrounding tissue and the surviving white matter tracts therein. A larger human ICH cohort analysis of the aforementioned parameters may provide robust objective surrogate markers of tissue injury.

## CONFLICTS OF INTEREST

The authors declare that they have no conflict of interest.

## Data Availability

Upon reasonable request, further data can be provided by the corresponding author Dr. Neeraj Chaudhary.
